# Retracted: Cardiac Arrest following a Myocardial Infarction in a Child Treated with Methylphenidate

**DOI:** 10.1155/2021/4712417

**Published:** 2021-01-28

**Authors:** Case Reports in Pediatrics

In the article titled “Cardiac Arrest following a Myocardial Infarction in a Child Treated with Methylphenidate” [1], the authors have re-evaluated the case in response to concerns that the conclusion that methylphenidate can cause cardiac arrest in otherwise healthy children was not supported. Since the publication of the article, the child passed away. The results of a genetic cardiomyopathy testing panel showed a lamin A/C mutation (heterozygote). With this knowledge, it is possible that the child suffered from cardiomyopathy rather than a remote myocardial infarction as suggested. The article is therefore being retracted with the agreement of the authors and the editorial board.
